# Comorbidities and inflammation associated with ovarian cancer and its influence on SARS-CoV-2 infection

**DOI:** 10.1186/s13048-021-00787-z

**Published:** 2021-02-25

**Authors:** Sima Chaudhari, Satyajit Dey Pereira, Meshach Asare-Warehene, Ritam Naha, Shama Prasada Kabekkodu, Benjamin K. Tsang, Kapaettu Satyamoorthy

**Affiliations:** 1 Department of Cell and Molecular Biology, Manipal School of Life Science, Manipal Academy of Higher Education, Manipal, Karnataka 576104 India; 2 Chronic Disease Program, Ottawa Hospital Research Institute and Department of Obstetrics & Gynecology and Cellular and Molecular Medicine, University of Ottawa, Ottawa, Ontario K1N 6N5 Canada

**Keywords:** Ovarian cancer, risk factor, SARS-CoV-2, Inflammation, Hormones

## Abstract

Coronavirus disease 2019 (COVID-19) caused by the novel severe acute respiratory syndrome coronavirus 2 (SARS-CoV-2) worldwide is a major public health concern. Cancer patients are considered a vulnerable population to SARS-CoV-2 infection and may develop several COVID-19 symptoms. The heightened immunocompromised state, prolonged chronic pro-inflammatory milieu coupled with comorbid conditions are shared in both disease conditions and may influence patient outcome. Although ovarian cancer (OC) and COVID-19 are diseases of entirely different primary organs, both diseases share similar molecular and cellular characteristics in their microenvironment suggesting a potential cooperativity leading to poor outcome. In COVID-19 related cases, hospitalizations and deaths worldwide are lower in women than in males; however, comorbidities associated with OC may increase the COVID-19 risk in women. The women at the age of 50-60 years are at greater risk of developing OC as well as SARS-CoV-2 infection. Increased levels of gonadotropin and androgen, dysregulated renin-angiotensin-aldosterone system (RAAS), hyper-coagulation and chronic inflammation are common conditions observed among OC and severe cases of COVID-19. The upregulation of common inflammatory cytokines and chemokines such as tumor necrosis factor α (TNF-α), interleukin (IL)-1β, IL-2, IL-6, IL-10, interferon-γ-inducible protein 10 (IP-10), granulocyte colony-stimulating factor (G-CSF), monocyte chemoattractant protein-1 (MCP-1), macrophage colony-stimulating factor (M-CSF), among others in the sera of COVID-19 and OC subjects suggests potentially similar mechanism(s) involved in the hyper-inflammatory condition observed in both disease states. Thus, it is conceivable that the pathogenesis of OC may significantly contribute to the potential infection by SARS-CoV-2. Our understanding of the influence and mechanisms of SARS-CoV-2 infection on OC is at an early stage and in this article, we review the underlying pathogenesis presented by various comorbidities of OC and correlate their influence on SARS-CoV-2 infection.

## Introduction

Since the first case reported in December 2019, coronavirus disease 2019 (COVID-19) has spread globally, resulting in the ongoing pandemic. Interestingly, the phenotypic symptoms of infected individuals are multi-systemic and diverse. While many display mild symptoms, some remain asymptomatic and may act as carriers. However, subgroups of the infected population may present severe phenotypes with acute respiratory distress and/or multi-organ failure [97]. These subgroups of patients exhibit comorbidities such as hypertension, diabetes, cardiovascular disease and respiratory disease [203]. The initial observation of poor outcome of cancer patients upon infection by severe acute respiratory syndrome coronavirus 2 (SARS-CoV-2), emphasises their increased risk. The immunocompromised state of cancer patients either because of the tumor pathophysiology or anticancer treatment was proposed as a contributing factor for enhanced susceptibility [102, 208]. However, cancer includes an assorted array of tumor of various organs and a recent study by Lee et al. [97] reported varied susceptibility of SARS-CoV-2 to different types of tumors [97]. Nonetheless, the mortality in cancer patients from COVID-19 is significantly correlated with gender, age and comorbidities rather than immunosuppression [96]. The comorbid conditions associated with COVID-19 mediates the hyper-inflammatory microenvironment that results in increased severity of the disease. In a study reported by Spezzani et al. [172] an immunocompromised metastatic breast cancer patient with COVID-19 had a quicker recovery time compared with an immunocompetent hypertensive COVID-19 patient, suggesting that comorbidities associated with hyper-production of cytokines are the key drivers of COVID-19 severity and hospitalization [96, 97, 172]. Age-related ovarian cancer (OC) and its associated comorbidities share similar molecular and cellular characteristics with the microenvironment of COVID-19 patients. Increased levels of gonadotropin and androgen [21, 46, 110] as well as dysregulated renin-angiotensin-aldosterone system (RAAS) are commonly reported in OC and severe cases of COVID-19. Additionally, inflammatory cytokines and chemokines such as interleukin (IL)-1β, IL-2, IL-5, IL-6, IL-7, IL-8, IL-10, tumor necrosis factor α (TNF-α), granulocyte colony-stimulating factor (G-CSF), vascular endothelial growth factor (VEGF), basic fibroblast growth factors (bFGF), macrophage colony-stimulating factor (M-CSF), growth-regulated oncogene alpha (GRO-α), interferon-γ-inducible protein 10 (IP-10), eotaxin, monocyte chemoattractant protein-1 (MCP-1) etc. (Table 1) are significantly upregulated and are common to both disease conditions. Further, hyper-inflammation and hyper-coagulation states appear to be common among these two conditions [114, 165]. These shared microenvironments suggest an increased risk of infection and severity of infection in OC subjects or altered risk of OC progression due to SARS-CoV-2.

**Table 1:** Significantly upregulated cytokines and chemokines in the serum of ovarian cancer and COVID-19 patients

	Immune mediators	Profile	References
**COVID-19**	Cytokines/Growth Factors	TNF-α, IL-1α, IL-1β, IL-2, IL-4, IL-5, IL-6, IL-7, IL-10, IL-12p40, IL-13, IL-16, IL-18, IFN-α2, IFN-γ, TRAIL, MIF, LIF, VEGF, SCGF, G-CSF, M-CSF, SCF, HGF, bFGF	[200, 201, 105, 106, 76, 203, 101]
	Chemokines	GRO-α, IL-8, MIG, IP-10, Eotaxin, CTACK, SDF-1α, MCP-1, MIP-1α, MCP-3	
**Ovarian cancer**	Cytokines/Growth Factors	TNF-α, IL-1β, IL-2, IL-5, IL-6, IL-7, IL-9, IL-10, VEGF, G-CSF, M-CSF, PlGF, PDGF-BB, bFGF	[60, 202]
	Chemokines	GRO-α, IL-8, IP-10, Eotaxin, MCP-1, MIP-1β, RANTES	

## Ovarian cancer

Ovarian cancer (OC) emerges from the ovaries [27] and/or the fallopian tube [95] with approximately 90% developing from the ovarian surface epithelium [27]. Based on their microscopic features, the epithelial ovarian cancers (EOCs) are further subtyped as serous (68-71%), mucinous (3%), endometrioid (9-11%), clear cell (12-13%), malignant Brenner (1%), and undifferentiated (6%) [183]. Using molecular characterization, EOCs are grouped into type I and type II cancers [184]. Mutations in oncogenes and tumour suppressor gene such as *KRAS, BRAF, PTEN, PIK3CA,* and *ARID1A* are relatively common in type I EOCs [161, 184]; however, mutations in *p53* are frequently reported in type II EOCs [138].

### Prevalence of ovarian cancer

OC is the most lethal gynaecological cancer predominantly due to late diagnosis, recurrence and chemoresistance [120, 134]. In 2018, the estimated age-standardized incidence and mortality rate of OC was 6.6 and 3.9 per 100,000 women respectively [17]. Transvaginal ultrasound coupled with CA125 detection are the conventional means of screening OC; however, their low-test accuracy present as a major setback in their clinical application [47, 207]. Although plasma gelsolin has shown promise as an early diagnostic biomarker in a smaller cohort of OC patients [8], it is yet to be validated in a larger cohort. This leans to the global efforts of developing novel diagnostic biomarkers to help in the early diagnosis of OC.

### Ovarian cancer risk factors and morbidities

OC is a multifactorial disease, and its diverse epidemiological characteristics are responsible for the disparities in its incidences across the world. Various comorbid conditions linked to OC include demographic, reproductive, gynaecologic, hormonal, genetic, and lifestyle factors. In addition, OC is linked with a plethora of risk factors including but not limited to age, obesity, unhealthy meals, smoking, alcoholism, early menarche, late menopause, null parity, hormone replacement therapy, mutations in *BRAC1/2*, and family history (Table 2) [134].

**Table 2:** Comorbidities and their association with ovarian cancer risk

Factors	Comorbidities	Association with ovarian cancer	Mechanism of pathogenesis explained by	References
Demographic	Age	Increased risk, Poor outcome	Gonadotropin theory, Inflammation theory	[25]
Reproductive	Ovulation	Increased risk	Incessant ovulation theory, Inflammation theory	[160]
	Pregnancy	Protective	Gonadotropin theory	[155]
	Pre-eclampsia Pregnancy	Increased risk	Inflammation theory	[20]
	Lactation	Protective	Gonadotropin theory	[132]
Gynaecologic	Pelvic inflammatory disease	Increased risk	Inflammation theory	[104]
	Endometriosis	Increased risk	Retrograde menstruation theory, Inflammation theory	[133]
	Tubal ligation	Protective	Inflammation theory	[123]
Hormonal	Oral Contraceptive Pills (OCPs)	Protective	Gonadotropin theory	[42]
	Hormone replacement therapy (HRT)	Controversial	Gonadotropin theory	[35]
Genetic	Family history	Increased risk		[88]
	BRCA mutation	Increased risk		[179]
	Lynch syndrome	Increased risk		[108]
Lifestyle	Food	Controversial	Inflammation theory	[146, 121]
	Adiposity/Obesity	Increased risk	Inflammation theory, Gonadotropin theory	[144]
	Smoking/ Caffeine	Controversial	Inflammation theory	[94, 119]
Environmental	Talc	Increased risk	Inflammation theory	[196]

### Ovarian cancer and basis for pathogenesis

Multiple risk factors are associated with OC; however, the underlying mechanism of pathogenesis remains to be fully understood. Based on evidence, several theories have been proposed which are - a) the incessant ovulation theory [44], b) retrograde menstruation theory [162], c) the gonadotropin theory [34] and d) the inflammation theory [140]. Among these, the gonadotropin and inflammatory theories are the most extensively explored.

#### Gonadotropin theory

Cramer and Welch in 1983 introduced the hypothesis of gonadotropin and suggested that a higher level of gonadotrophin may contribute to the pathogenesis of OC; however, the suppression of pituitary gonadotropin secretion may decrease the risk of OC [34]. Endocrine hormones such as follicle stimulating hormone (FSH) and luteinizing hormone (LH), are the important regulators of gametogenesis and steroidogenesis [27]. During ovulation, pregnancy, lactation, or menopause, the levels of these hormones are significantly altered. The damage associated with the rupture of follicle during ovulation resulting from high levels of FSH, LH and estrogen are also risk factors and may lead to OC. The higher levels of progesterone along with minimal estrogen production are associated with increased protection against OC. Prolonged production of progesterone during pregnancy interrupts the ovulatory cycle reducing the frequency of damage caused by ovulation [27, 129, 155, 160]. Oral contraceptive pills mostly entail a combination of estrogen and progesterone and act by inhibiting gonadotropin production leading to ovulation suppression [42]. Hormone replacement therapy with estrogen alone fails to improve in the survival of OC patients but its combination with progesterone reduces the risk of OC [157]. During lactation, the suckling effect limits the secretion of estrogen thereby preventing the normal pulsatile secretion of LH; an effect that result in anovulation [124]. Taken together, these findings suggest that increased gonadotropin production could be possibly implicated in OC pathogenesis.

#### Inflammation theory

Inflammatory mediators are crucial for the maintenance of normal physiological homeostasis. However, the imbalance between pro- and anti-inflammatory mediators may result in self-tissue damage leading to the risk of mutagenesis and aberrant cell growth [1]. Pro-inflammatory mediators may contribute to ovarian cancer by a) direct effect on tissues promoting transformation and b) indirect effect where the inflammation aid in the migration of malignant cells from other regions within the organ [167, 204].

#### Direct effect

Epithelial cells, stromal fibroblast, and leukocytes in the female reproductive tract highly express immune components including pattern recognition receptors [toll like receptors (TLRs)] and secreted molecules such as antimicrobial peptides [human neutrophil peptide (HNP1); lysozyme, lactoferrin], cytokines (IL-1, IL-6), and chemokines [CC-chemokine ligand 2 (CCL2)]. These immune components show spatio-temporal variation within the reproductive tract and their distribution is critically altered by the changes in hormone secretion during different stages of the ovulation cycle [197]. In OC, the dysregulated secretion of cytokines such as IL-1, IL-2, IL-6, M-CSF, TNF-α and others [9] as well as elevated tumor necrosis factor receptor 1 (TNFR1) [83] are observed, suggesting contribution of inflammation during tumor progression. Inflammatory cytokines create a microenvironment that promote DNA aberration and mutagenesis (*via* a nitric oxide dependent mechanism) [81], inactivate the tumor suppressor genes such as *p53* and enhance cell proliferation and oncogenesis by the activation of hypoxia-inducible factor 1- alpha (HIF1α), nuclear factor kappa B (NF-κB), and/or signal transducer and activator of transcription (STAT) 3 pathways [58, 65, 69, 70, 74, 77, 165, 166]. During ovulation, inflammatory mediators including leukotrienes and prostaglandin as well as vasoactive agents such as bradykinin induces the inflammatory reaction [36].

The association of metabolic disorders such as type 2 diabetes and obesity with poor outcomes of EOC has been lucidly reviewed by Craig et al. [33]. The pathological factors for these observations have been attributed to the altered expression profiles of cytokines and adipokines. Such changes induce altered immune responses towards the proliferation of tumor cells as well as pro-tumorigenic signaling pathways [14, 33, 98]. Women with higher adiposity and a higher level of C-reactive protein (CRP) and IL-6 are suggested to be more prone to the incidence of OC [144]. *In vitro* studies have demonstrated the ability of estrogen to stimulate B cell response and depletion of suppressor T cells contributing towards the elevated antibodies and autoantibodies level [118], hence increasing the risk of OC [35]. The increased risk of OC among the individual with high intake of red meat and processed meat is because of increase in the source of iron, salt, saturated fats, and other factor associated with DNA damage such as heterocyclic amines, nitrosamine and N-nitroso compounds and polycyclic aromatic hydrocarbon [5, 23]. In contrast, the reduced risk upon consumption of a plant-based diet was associated with a reduction in cancer-promoting hormones [112].

#### Indirect effect

Although ovulation has a significant contribution towards the etiology of OC, the incidence of EOC is higher in the women of 63 years old who have attained menopause [55]. During post-menopause, the ovarian follicles are predominantly depleted with the remaining ovary being reduced to collagenous scar tissue [86]. The migration of malignant cells from oviduct to ovary *via* cytokine/chemokine gradient formed from the surface of the wounded ovary potentially explains why menopause women are at a higher risk of OC [86, 204]. This phenomenon might also explain the protective effect of hysterectomy and tubal ligation [62, 86] against the development of OC.

## SARS-CoV-2 and Inflammation

SARS-CoV-2 is a novel coronavirus which causes COVID-19, a disease that has been denoted pandemic globally by the World Health Organisation (WHO). SARS-CoV-2 shares about 80% genomic similarity with SARS-CoV which was responsible for a respiratory disease outbreak in 2003 [24]. As of 28^th^ January 2021, the WHO had reported 99,864,391 confirmed COVID-19 cases and 2,149,700 deaths worldwide [199].

Most of our current understanding of inflammatory effects of SARS-CoV-2 comes from several studies related to lung injury and respiratory distress. SARS viruses cause acute respiratory distress syndrome (ARDS) and acute lung injury (ALD). The alveolar cavities of SARS patients contain desquamated epithelial cells, some of which are large and consist of syncytial nuclei [66]. The syncytial nuclei represent the cytopathogenic effect caused by viral replication in the cells [164]. Immunostaining of pulmonary interstitial tissues demonstrates the infiltration of monocytes, lymphocyte, and macrophages, fibrin deposition, formation of the hyaline membrane, and fibrinosis of alveolar extrude. Besides, ACE2 expressing cells infected with SARS-CoV demonstrated a higher level of transforming growth factor-beta 1 (TGF-β1) and MCP-1 with moderate levels of IL-1β, IL-6, and TNF-α. Thus, the histological changes and the upregulation of pro-inflammatory cytokines in the cells of patient with SARS-CoV, reconcile the acute lung injury [66]. MCP-1 is a chemotaxis promoting factor which induces macrophages migration towards the affected sites [205]. Upon stimulation with several pro-inflammatory cytokines, macrophage undergoes proliferation, or are activated to produce more pro-inflammatory cytokines [143]. TGF-β1 enhances Fas-mediated cell apoptosis leading to the death of alveolar epithelial cells, an outcome resulting in acute lung injury [64]. Although the production of inflammatory cytokines in SARS-infected cells are initiated for combating the invading virus, the hyper-production of inflammatory cytokines might attack both infected as well as uninfected cells, culminating in significant ARDS and ALD [66]. An increase in pro-inflammatory cytokines has also been observed in other SARS, including acute exacerbation of chronic obstructive pulmonary disease and avian influenza (H5N1), suggesting overexpression of inflammatory cytokine is the common mediator of ALD and ARDS in a patient infected with a virus *via* respiratory pathway [26, 213].

In contrast to non-severe SARS-CoV-2 infected patients, an abundance of cytokines and chemokines such as MCP1, G-CSF, IP10, TNF-α, and macrophage inflammatory protein 1alpha (MIP1A) were reported in severe cases [76]. Indeed, among the deceased patient of COVID-19, the level of TNF-α, IL-6, IL-1β, and IL-8, were the highest when compared to recovered cases [115]. The COVID-19 patient with ARDS also shows increased chemokine receptor 6 (CCR6)^+^ T helper 17 (Th17) cells which are derived from CD4^+^ T cells [200, 201]. Th-17 cells induce IL-17, IL-21, IL-22, and GM-CSF [111]. There was a significant correlation of IL-17 with the lung injury and is one of the pro-inflammatory cytokines capable of producing a vast number of inflammatory chemokines and cytokines [18, 130].

## Microenvironment shared by SARS-CoV-2 and OC affected individuals

Although SARS-CoV-2 and OC affect distinctive primary organs, the molecular signatures in their respective microenvironment seem to be common. This suggests a common mode of pathogenesis and also supports a) the altered risk of the OC progression in COVID-19 patients, b) augmentation of infection by SARS-CoV-2 in OC patients and c) higher risk of virus infection in individuals with comorbidities associated with OC. OC and COVID-19 patients report chronic inflammation and hyper-coagulation which could be the result of a dysregulated RAAS system [114, 165, 174]. Further, the levels of gonadotropin and androgen are also higher in both disease conditions [21, 46, 110]. Whether RAAS and hormonal regulation predispose OC patients to COVID-19 is the subject of future investigation.

### Physiological Role of Renin-Angiotensin-Aldosterone System (RAAS)

RAAS is an essential regulatory mechanism for blood pressure and homeostasis. It is inclusive of two functionally opposing axes- ACE/Ang II/AT1 (proliferative/ vasoconstrictor) and ACE2/Ang-(1-7)/MAS1 (anti-proliferative/ vasodilator) [114]. The precursor protein angiotensinogen secreted from the liver forms angiotensin I (Ang I) when cleaved by renin. ACE then converts Ang I to Ang II. Alternatively, ACE2 can act on Ang I to form angiotensin 1-9 [Ang-(1-9)]. Furthermore, ACE can convert Ang-(1-9) to Ang-(1-7) while Ang II can be converted to Ang-(1-7) by ACE2 [195]. The successive action of neutral endopeptidase (NEP) and prolyl endopeptidase (PEP) on Ang I also lead to the formation of Ang-(1-7) [6]. Binding of Ang II to ATR1 trigger downstream signaling pathways which involves a) phosphorylation of p65 subunit of the transcription factor, NF-κB, leading to the release of cytokines such as IL-1β, IL-6, TNF-α and IL-10 [82, 114, 170], b) activation of mitogen-activated protein kinase (MAPK) pathway to release IL-1, TNF-α, IL-10 and IL-12 [7, 114, 125], and c) tans-signaling of IL-6/ soluble interleukin 6 receptor (IL-6/sIL-6R) complex that results in activation of STAT3 *via* glycoprotein (gp130) [31, 114]. Thus, activation of the ACE/Ang II/AT1R axis induces a continuous cascade of inflammatory responses [114]. To overcome the activity of ACE/Ang II/AT1R axis, the ACE2/MasR axis of the RAAS system is initiated when Ang II is converted to Ang-(1-7) by ACE2. Antagonistic to Ang II, Ang-(1-7) is a vasodilatory peptide with anti-thrombotic, anti-proliferative, and anti-inflammatory activities. Binding of Ang-(1-7) to MasR receptor suppresses NF-κB, p38-MAPK, and expression of inflammatory proteins thus preventing the damage [177, 209, 216]. Additionally, Ang-(1-7) modulates the extracellular signal-regulated kinase 1/2 (ERK1/2) pathway by phosphorylation of ERK1/2 in the β-arrestin dependent mechanism. ERK1/2 is involved in the regulation of anti-inflammatory cytokine IL-10. IL-10 enhances the differentiation of T helper (Th) cells to Th2 type, which in turn produces anti-inflammatory cytokines such as IL-4, IL-5, IL-9, and IL-13 [48, 114, 175].

Two other G-protein coupled receptors which are involved are bradykinin B1 receptor (BKB1R) and bradykinin B2 receptor (BKB2R). While the vasodilator bradykinin (BK) is the ligand for BKB2R, the agonist of bradykinin (des-Arg9)-BK/ DABK is the ligand for BKB1R [116, 117]. Although in normal physiological conditions, expression of BKB1R is negligible, its expression is upregulated by cytokines such as TNF-α and IL-1β. BKB1R activation enhances the neutrophil infiltration and increases the level of IL-1B and MCP-1 [16, 38, 122, 150]. The ligand of BKB1R, DABK, which is also an inflammatory factor, is normally cleaved by ACE2 to deactivate the DABK and prevent the release of inflammatory cytokines [114, 186].

Although earlier identified as involved in the maintenance of blood pressure and homeostasis, the importance of RAAS system in the ovaries of mammal inclusive of human have been demonstrated. The ovary is the major source for the precursor of renin, prorenin [147]. Ang-(1-7) is considered important for steroidogenesis, and the release of LH-stimulated progesterone was prevented by inhibition of ACE and PEP [149]. In the active ovary, the mRNA expression of MAS, ACE2, and ACE was observed in the granulosa cell. Additionally, the theca cells of secondary follicles were positive for Ang-(1–7) and MAS in the ovulatory stage while, it was negative in pre-ovulatory follicles. The staining of stromal cells of follicles showed the presence of MAS receptor, ACE2, and Ang-(1-7); but the Ang-(1-7) concentration was significantly lower in comparison to Ang II. This may be due to the short half-life of Ang-(1-7) in the plasma [40, 154].

The alteration in the expression level of various RAAS components is suggested to be regulated by the gonadotropin [40]. The patients treated with recombinant FSH or human chorionic gonadotropin (hCG) reported increased plasma Ang-(1-7). Ang II receptors are expressed in theca and granulosa cells. The increased level of Ang II has been observed after the LH surge [2]. The*  in* *vivo* treatment with gonadotropin-releasing hormone (GnRH) reportedly increased the expression of both Ang II and Ang-(1-7). Tonellotto dos Santos et al. [178] demonstrated that continuous expression of PEP, NEP, ACE2, and MAS in granulosa and theca cells after treatment GnRH; however, Ang-(1-7) level was elevated at 24hours post-treatment. This elevation in Ang-(1-7) was suggested as a result of cleaved Ang II rather than the conversion of Ang I to the intermediate peptide [178].

### Effect of dysregulated RAAS on SARS-CoV-2 and OC

#### Cytokines overproduction

Respiratory transmission is the primary route of infection for SARS-CoV-2 [56]. The entry of SARS-CoV-2 into the host cell is mediated by binding of its surface spike glycoprotein (S protein) to the angiotensin-converting enzyme 2 (ACE2) receptor of the host cell, however; ACE2 is minimally expressed in human lung cells [56]. Interrogating microarray datasets of previous infections of Middle-East respiratory syndrome coronavirus (MERS-CoV) and SARS-CoV indicated increased mRNA expression of ACE2. Similar observations have been made during the ongoing pandemic where ACE2 expression at transcript level was 3.6 fold higher in the nasopharyngeal swab specimens from COVID-19 positive patients [215]. ACE2 upregulation is mediated by an innate immune signaling pathway activated by TLR and RNA sensor (retinoic acid-inducible gene I /melanoma differentiation-associated gene 5- mitochondrial antiviral signaling protein axis or RIG-I/MDA-5-MAVS signaling cascade), which ultimately induce the production of inflammatory cytokines [215]. Other studies have suggested an alternative mechanism for inflammatory cytokine production where cyclic GMP-AMP synthase-stimulator of interferon genes (cGAS-STING) mediate the activation of NF-κB signaling cascade [141]. The promoter of ACE2 contain a binding site for various transcription factors, including STAT5, STAT1, STAT3, c-Jun, interferon regulator factor (IRF)1, IRF8, and IRF2 [215]. Human bronchial epithelial cells treated with interferon-beta (IFN-β) showed a significant increase in ACE2 mRNA expression. ACE2 promoter activity was also enhanced by inflammatory cytokines such as interferon-alpha (IFN-α), IFN-β, interferon-gamma (IFN-γ), TNF-α, IL-6, IL-1α, or IL-1β [215]. Expression of the membrane ACE2 receptor is a critical factor for the initiation and spread of infection by SARS-CoV and SARS-CoV-2 [185, 190]. The release of interferon and cytokines to the surrounding cells by virus-infected cells might enhance the expression of ACE2 in nearby uninfected cells, thereby rendering them susceptible to SARS-CoV-2 infection.

Although mRNA expression of ACE2 increases upon SARS-CoV infection, it is downregulated at the protein level implying a possible truncation or secretion upon infection [57]. The absence of membrane bound ACE2 may inhibit the formation of Ang-(1-7), thus disturbing the ACE2/Ang1-7/MAS-axis function [54, 100]. Whether this phenomenon is recapitulated in SARS-CoV-2 infections as observed in SARS-CoV infections will remains to be determined. COVID-19 patients are characterized by excessive cytokine production, pulmonary shutdown, and thromboembolism events. Imbalance in the ACE/ACE2 pathway results in hypertension, atherosclerosis, thrombosis, heart or kidney failure, and severe respiratory distress, the comorbidities observed among COVID-19 positive patients [52, 163]. This suggests that dysregulation in the function of angiotensin-converting enzyme-1/angiotensin II/ angiotensin II type 1 receptor (ACE1/Ang-II/AT1) axis and angiotensin-converting enzyme-2/ Angiotensin (1-7)/ MAS1 proto-oncogene (ACE2/Ang1-7/ MAS) axis play a critical role in the pathogenesis of COVID-19.

The disorderly release of cytokines, also known as cytokine storm, leads to a hyper-inflammatory state and have been previously associated with the severity of SARS-CoV and MERS-CoV infections as well as now with the SARS-CoV-2 [89, 171, 198]. The downregulation of ACE2 upon infection is considered the underpinning pathology behind the disease’s severity as recombinant ACE2 protects mice from acute lung failure [78]. Treatment with ACE2 neutralized the virus by competitive binding. Thus, the cellular ACE2 remains available for maintenance of the counter-regulatory function.

The binding of SARS-CoV-2 to the host cell receptor triggers receptor-mediated endocytosis of the complex, leading to downregulation of ACE2 [54]. This downregulation of ACE2 potentially results in a continuous supply of Ang II in the presence of unaltered ACE while decreasing the levels of Ang-(1-7). Elevated levels of Ang II in the serum was reported in COVID-19 subjects, and the level correlates with the severity of the infection [105, 106]. Additionally, increased differentiation of CD4^+^ lymphocytes to Th-17 was demonstrated with an increase in Th-17 response in MERS-CoV, SARS-CoV, and SARS-CoV-2 patients [105, 106, 114]. Inhibition of ACE in COVID-19 patients with hypertension reported reduced disease severity with downregulation of IL-6 and higher T cell numbers, suggesting the exaggerated activation of Ang-II/AT1R axis may contribute towards the uncontrolled cytokine response leading to ALD [114, 127]. Similarly, in the presence of inflammatory cytokines, expression of BKB1R is enhanced while, the absence of ACE2 result in activated DABK which, when bound to its receptor BKB1R, initiates the inflammatory signaling producing more cytokines [114, 150, 186]. Thus, downregulation of the ACE2 after SARS-CoV-2 infection deregulates the RAAS, attenuates Mas receptor (ACE2/MasR axis), and activates [des-Arg9]-bradykinin (ACE2/bradykinin B1R/DABK axis) and these are responsible for the overproduction of cytokines causing the cytokine storm [114].

Another pathway that contributes toward a cytokine storm is the activation of complement cascade upon viral infection. Previous *in vivo* mice study for acute respiratory viral infection (MERS-CoV and SARS-CoV), reported hyperactivation of complement component (C)5a and C5b in serum and lung respectively. The mice model deficient for C3 were mildly infected by SARS-CoV and had less circulatory chemokines and cytokines, suggesting viral infection activates the complement cascade that induces inflammatory process [61, 87]. Investigation of the lung of deceased COVID-19 patient reported deposition of C3, C3a, as well as complement complex C5b-9 in the lung and elevation of C5a in the circulation [113]. C5a is the potent mediator of inflammation that can increase secretion of IL-6, TNF-α, and IL-1 from TLR-2, TLR-4, and/or TLR-9 stimulated macrophages [43, 114]. The complement cascade’s activation is suggested to be mediated by auto-activation of the key serine protease in the lectin pathway upon binding of the viral nucleocapsid (N) to mannose-binding lectin (MLB), the intermediate of MLB complementation pathway [50]

In the context of ovary, although normal follicle’s stromal cells showed intense staining for Ang-(1-7), MAS and ACE2 receptor, their expression in the postmenopausal women was less intense. ACE activity is comparatively higher in the ovary of postmenopausal than premenopausal women [40, 154]. In normal and ovarian tumors, AT1 is localized in the cytoplasm but its expression was evident only in the OC correlating with the stage of cancer [79]. The overexpression of angiogenic VEGF and increased micro-vessel density, along with AT1 overexpression in OC patients with poor outcomes, suggested the role of dysregulated RAAS in OC development [174]. A recent study by Zhang et al. [211] revealed the triggering of classical ATR1 pathway (through AKT and ERK signaling) by Ang II and also by the transactivation of epidermal growth factor receptor (EGFR) pathway. Further ATR1 activation upregulates stearoyl-CoA desaturase-1 (SCD1) which promotes progression and metastasis as SCD1 increases lipid desaturation, thus reducing the stress to the endoplasmic reticulum [211].

#### Coagulation

A higher level of mean platelet volume (MPV) and observation of thromboembolism among the COVID-19 patient [210] suggest a dysregulated coagulation pathway. Du et al. [41] suggested the downregulation of ACE2 expression as well as a dysregulated immune response because of cytokine storm can lead to vascular injury and activation of the platelets. Similarly, increased production of other factors of coagulation pathways such as tissue factor, plasminogen, and others lead to a hypercoagulable state resulting in the thromboembolic events, as reported in a severe case of COVID-19 [41].

Microarray based gene expression profiling of peritoneal structure in individuals with EOC showed altered expression of inflammation and coagulation associated genes [188]. The observation of thromboembolism among cancer patients could be due to the activation of the coagulation cascade [156]. The coagulation pathway proteins have also been identified to contribute to tumor cell proliferation, invasion angiogenesis, and metastasis [193].

## Influence of OC microenvironment to SARS-CoV-2 infection

**Fig. 1:**
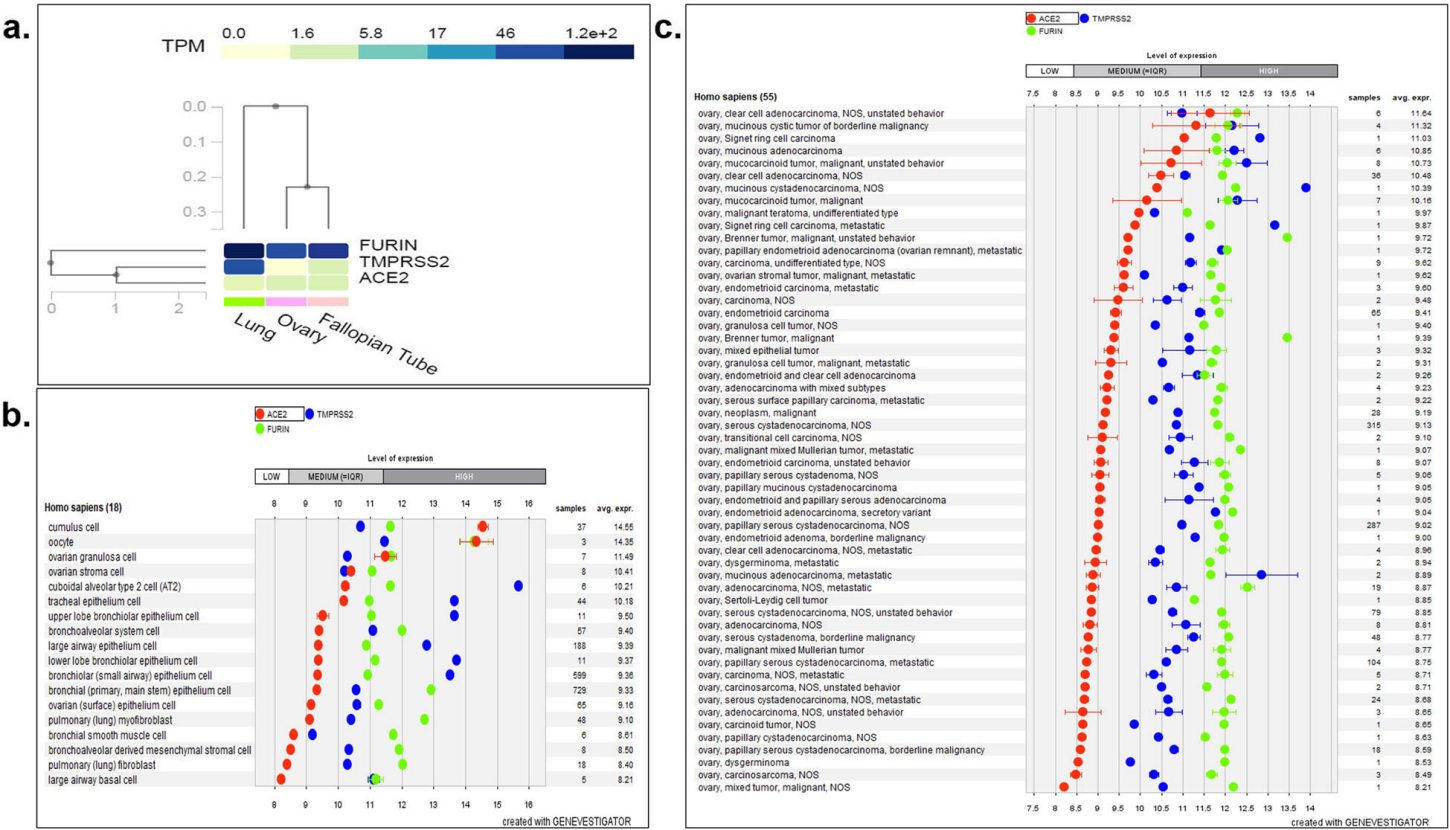
*In silico* gene expression profiles in various tissues. Expression of *ACE2*, *TMPRSS2*, and *FURIN* genes in (**a**) normal ovarian, lung, and other female reproductive organ tissues (data accessed *via* GTEx portal), (**b**) various normal cells of lung and ovary and (**c**) in ovarian and fallopian neoplasms (created using Genevestigator). Analysis revealed medium to high expression of the genes in the malignant ovarian tissues suggesting a potential route of SARS-CoV-2 entry

Compared to females, males are considered more susceptible to the SARS-CoV-2 infection and one of the factors suspected for the susceptibility is the differences in the sex hormone [52]. However, with an increase in age, this difference becomes less evident. Although the FSH and LH levels are altered over various phases of the menstrual cycle, the concentration remains higher among the elderly women. The highest concentration is among the women who had attained menopause as they lack negative feedback mechanisms by ovarian steroids [19, 21]. LH induces the synthesis of androgens while FSH stimulates estrogen synthesis from the androgen [53]. However, in the absence of pre-ovulatory follicles during menopause, estrogen cannot be produced; thereby, an increase in the androgen level can be expected. Fogle et al. (2007) [46] has demonstrated a significant contribution of the postmenopausal ovary in the production of testosterone and androstenedione [46]. Estrogen is considered as an immune stimulant when present at a basal level and as an immune suppressive hormone at a higher level [52, 173]. Menopause women are reported with higher TNF-α, IL-6, and CRP levels [168]. Thus, the low level of estrogen can be considered as one of the contributors to create an inflammatory microenvironment among postmenopausal women. Cannon et al. [21] reported enhanced secretion of cytokines IL-1β, IL-6, TNF-α and upon *in vitro* treatment of monocytes with exogenous FSH [21]. LH depletes the antioxidant, ascorbic acid, thus increasing the free radical levels and inflammatory mediators [140]. Since ACE/ACE2 increases with the age; the RAAS pathway imbalance can lead to excessive cytokine and chemokine production thus contributing to a higher inflammatory response observed among menopausal women [145, 206]. Several proteases are reported to activate coronaviruses *in vitro*, including trypsin-like serine proteases such as the transmembrane serine protease (TMPRSS)2, TMPRSS11A, TMPRSS11D, FURIN and cathepsin L [11, 67, 71, 131]. *FURIN* and *TMPRSS2* are reported to be candidate genes relevant for SARS-CoV-2 entry into the host cells [72]. Expression of TMPRSS2, which is involved in priming of S-protein during SARS-CoV-2 infection is stimulated by several pro-inflammatory conditions as well as androgens [56]. It is therefore conceivable that individuals with OC and comorbidities could be more susceptible to SARS-CoV-2 infection. *In silico* expression analysis of *ACE2, TMPRSS2,* and *FURIN* genes in normal ovarian and fallopian tube tissue and the constituent cells were carried out using Genotype-Tissue Expression (GTEx) [22] and Genevestigator [75] respectively. Further, the expression levels were compared to that in lung tissue. A consistently high expression of *FURIN* across the tissues and constituent cells was observed whereas, *ACE2* expression levels across the tissues and cells were similar in both lung and ovary. *TMPRSS2* showed high expression levels in the lungs and medium to low expression in ovary and fallopian tissue (Fig. 1a, b). The expression levels of these three genes when analyzed in different subtypes of ovarian carcinomas using Genevestigator showed medium to high expression in the malignant tissue (Fig. 1c). This suggests a greater susceptibility of viral entry in ovarian tumors.

## Influence of SARS-CoV-2 infection on OC

The circulatory inflammatory marker specifically IL-2, IL-4, IL-6, IL-12, and IL-13 are associated with the risk of EOC [30]. Cytokine storm (hyper-production of inflammatory cytokines) during SARS infection could increase the risk of OC. While interaction of the pro-inflammatory cytokine IL-6 with its membrane-bound receptor activates classical IL-6 signaling and contributes to its role in inflammation, its interaction with sIL-6R mediates the function of pro-inflammatory cytokines *via* trans-signaling pathway [153]. Increased expression of sIL-6R and IL-6 are reported in malignant ascites of OC patients. The IL-6/sIL-6R complex has been demonstrated to activate ERK pathway on endothelial cells and increases endothelial hyper-permeability *via* Src kinase activation and phosphorylation of vascular endothelial-specific cadherin (VE-cadherin). Thus, IL-6 trans-signaling by sIL-6R contributes toward endothelial survival, migration, and integrity resulting in the progression of OC [107]. In patients infected with Influenza A virus (IAV) infection, there is an elevation of soluble IL-6R, an outcome that increases the expression of its ligand, IL-6 and inflammatory cytokine IL-32. This suggests that sIL-6R is a key molecule involved in inflammatory response to viral infection [191]. Similarly, concomitant expression of sIL-6R during SARS-CoV-2 infection could potentially create a microenvironment in the ovary or distant organs to initiate the cancer progression.

**Fig. 2:**
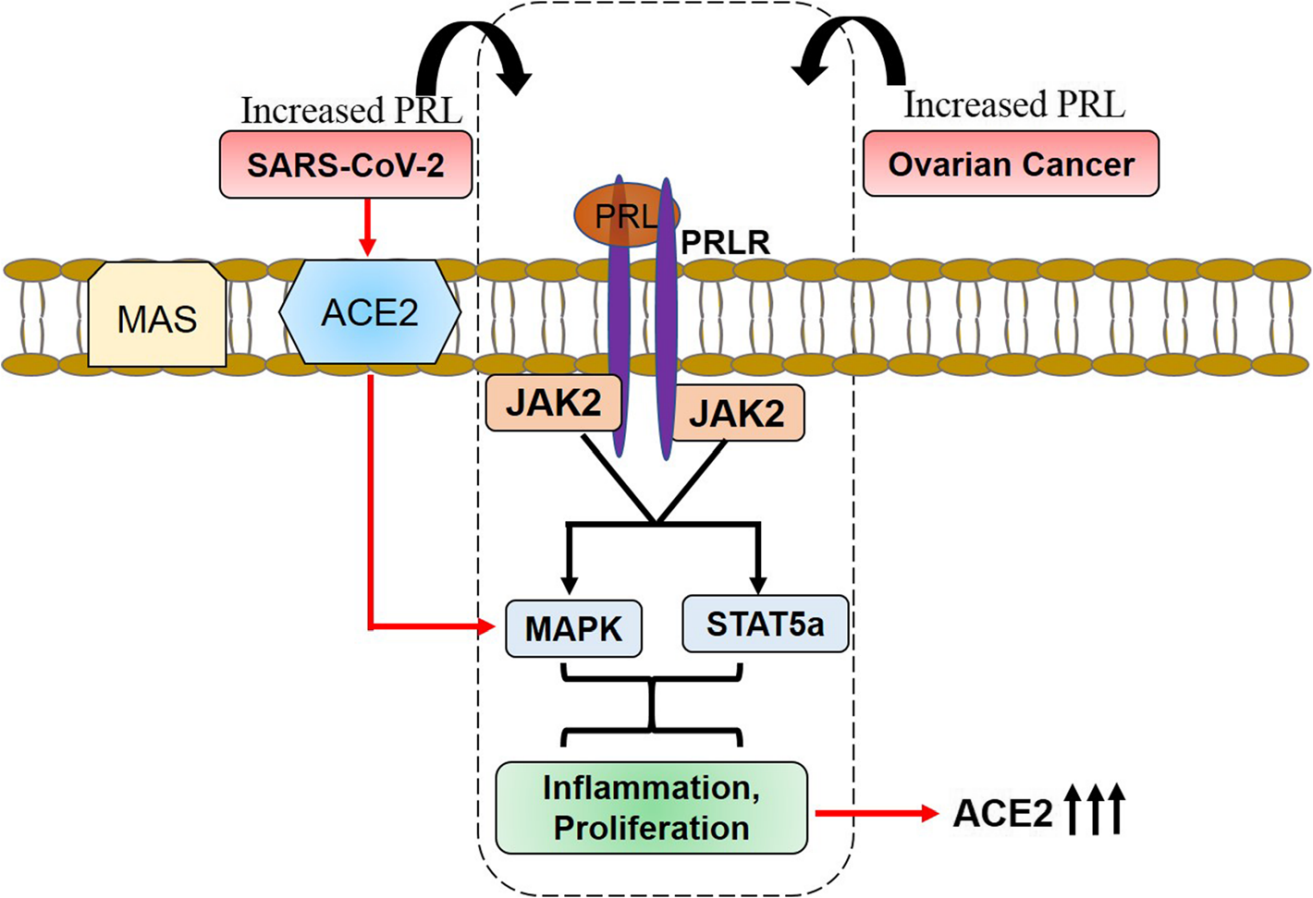
The influence of serum prolactin levels in both COVID-19 and OC affected subjects. Competitive inhibition of ACE2/Ang-(1-7)/Mas axis due to viral binding can inhibit ACE2 mediated suppression of MAPK. Hence, SARS-CoV-2 binding to ACE2 receptor may increase MAPK activation which can be further potentiated by PRL binding to PRLR thereby substantially increasing the MAPK activity in the cells leading to increase proliferation and inflammation, hence assisting in severe outcomes of ovarian cancer

The serum profiling of both ovarian cancer individuals as well as SARS-CoV-2 patients reported an elevated level of prolactin (PRL) [99, 110]. PRL binding activates prolactin receptor (PRLR), a type-1 family cytokine receptor, which then stimulates a signaling cascade through the activation of STAT5 [4, 68] resulting in increased inflammation and proliferation [181]. PRLR cascade can activate MAPK pathway [3], which is reported to be inhibited by the ACE2/Ang-(1-7)/Mas axis [128]. Competitive inhibition of ACE2 receptor through viral binding can downregulate the ACE2/Ang-(1-7)/Mas axis, thereby stimulating MAPK activity (Fig. 2). These suggest a possible role of PRL in potentiating severe outcomes of OC in COVID-19 subjects.

## Increased severity of SARS-CoV-2 infection in ovarian cancer patients

Upon infection, the innate immune signaling is activated which can be mediated by RIG-1/MDA5-MAVS axis [136]. RIG1 like receptors (RLRs) are pathogen recognition receptors that are activated upon recognition of the pathogen-associated molecular patterns (PAMPs) and these interact with MAVS to form a complex. This complex activates TANK binding kinase 1 (TBK1) and inhibitor of nuclear factor kappa-B kinase subunit epsilon (IKKε) and phosphorylates transcription factor IRF3 [136]. Alternatively, cGAS-STING pathway exists in which the cyclic dinucleotides species (CDNs) such as c-di-AMP or cyclic GMP-AMP (cGAMP) are produced by cGAS upon association with cytosolic double-stranded DNA or double-stranded RNA [29]. The association of activated STING with TBK1 at the Golgi complex is important to activate NF-κB and IRF3 to produce type I interferon and inflammatory cytokines [142]. However, the non-canonical pathway in the cGAS-STING pathway also exists which activates NF-κB in TBK1 independent manner [10, 152].

Type 1 interferon levels are minimal in patients infected with SARS-CoV-2. Additionally, non-structural protein (NSP)13 and NSP15 interact with TBK1 or its adaptor protein [59] although TBK1 phosphorylation is inhibited [13, 59]. These findings suggest inhibition of RIG-1/MDA5 axis by blocking the activation of TBK1 upon viral infection. However, the cytokine levels and transcriptome profiling from the cell lines infected with SARS-CoV-2, showed cGAS-STING mediated activation of NF-κB and inflammatory response [141]. Activation of cGAS occurs upon sensing the released DNA from mitochondria or nuclei due to cellular stress rather than direct sensing of dsRNA of SARS-CoV-2 [73]. The dsRNA is formed during replication and in case of SARS-CoV2, replication occurs within the double membrane vesicle formed during endocytosis [92, 93]. Neufeldt et al., [141] suggested the translocation of activated STING from ER to Golgi is inhibited by SARS-CoV-2 infection, thereby, inhibiting its interaction with TBK1 and its downstream pathway. However, activated STING stimulates NF-κB by a non-canonical pathway to produce inflammatory cytokines [141].

In various OC cell lines and tissue, impaired expression of cGAS and STING have been reported which is mediated by methylation of the promoter region [37]. Although the RIG-1/MDA5 axis is intact, the ability of SARS-CoV-2 to block this axis could suggest a potential increase in severity upon infection of the OC patients with SARS-CoV-2 because of the suppression of innate immune signaling pathway [37]. Besides, cGAS-STING activated signaling may also lead to inflammation-induced carcinogenesis and hence may worsen the disease.

## COVID-19 mRNA vaccine, inflammation and effects on ovarian cancer subjects

Several curative strategies such as combination treatment with systemic corticosteroids (dexamethasone), oxygen therapy, convalescent plasma from recovered COVID-19 patients, drugs such as tocilizumab, hydroxychloroquine, and remdesivir were deployed against SARS-CoV-2 as no definitive vaccine and vaccination strategy were available [12]. However, their impact on individuals with OC or any other cancer types remains to be established. We highlight briefly below the potential vaccine effects on effector cells and molecules associated with inflammation and OC.

During viral replication after host cell entry, an infected cell undergoes programmed cell death leading to release of damage associated molecular patterns (DAMPs). These molecules are recognized by macrophages, nearby epithelial and endothelial cells and are triggered to release inflammatory chemokines and cytokines. This initiates a cascade of inflammatory reactions where macrophages, monocytes, and T cells are attracted to the site of infection [176]. Naive CD4+ T cells are presented with viral antigens by antigen presenting dendritic cells and are further activated to release IL-2, TNF-α, and IFN-γ. CD4+ T cell promotes differentiation of cytotoxic CD8+T cells *via* the secretion of cytokines. Upon activation of these T cells through antigen recognition, the cells undergo clonal expansion [148] and eliminate the virus infected cells through several well established mechanisms which induce apoptosis or release of cytokines such as TNF-α and IFN-γ [90, 180]. Specific CD4+T cells elicit potent B cell responses that result in antibody affinity maturation [84]. SARS-CoV-2 specific CD4+T cells and CD8+T cells have been identified in the recovered COVID-19 patients and these are shown to recognize peptides of viral spike, nucleoprotein, and matrix as well as other viral proteins [32, 63].

Based on above immune response strategies, development of vaccine against COVID-19 had been initiated. Two mRNA-based SARS-CoV-2 candidate vaccines, mRNA-1273 [187] and BNT162b2 [80] were the first to be granted emergency authorization in Europe and USA. Both are composed of nucleoside modified mRNA encoding full length prefusion SARS-CoV-2 spike protein encapsulated in lipid nanoparticles [182]. The data provided after Phase 1/2 study for BTN162b2 reported robust expansion of spike protein specific CD4+ and CD8+ T cell responses. Cytokines such as IFN-γ and IL-2 were produced and CD8+ T cell responses against multiple regions of spike protein were molecularly identified [159]. Previously, vaccination with human papillomavirus (HPV) L1 virus-like particles (VLP) have demonstrated to induce dendritic cells and potent B cells, hence induction of cytokines such as IL-2, IL-4, IL-5, IL-6, IL-10, IL-13, TNF-α, IFN-γ, IP-10, and MIP-1 [51]. Similarly, influenza specific CD4+ and CD8+ T cell response as well as release of cytokines such as IL-5, IL-9, IL-10, IL-13, IL-17A, IL17F, IL-21, and IFN-γ was reported upon administration of virus like particle based vaccine [169]. However, extensive data on the efficacy and safety of the COVID-19 mRNA vaccine on an individual with comorbid conditions and cancer remains to be reported.

The severe cases of COVID-19 are often presented with cytokine storm and lymphopenia [76, 203]. The severely ill and deceased COVID-19 subjects show expressively lower lymphocyte (CD8+ and CD4+ T cells) levels than the survivors [39, 158]; however, with the higher neutrophil counts compared to lymphocytes [103, 189]. In addition, the CD4+ and CD8+ T cells from critically ill COVID-19 patients highly express inhibitory receptor such as T-cell immunoglobulin mucin-3 (Tim-3) and Programmed cell death protein 1 (PD-1) [109, 212, 214]. The increased level of TNF-α, IL-6, and IL-10 is inversely correlated with the decreased T cell population [85, 135] and IL-2, IL-7, IL-15, and IL-21 were reported to upregulate PD-1 expression on T cells [91]. Expression of these markers indicate exhaustion of T cells with decreased cytokine production and cytotoxic function [109]. The inefficiency of immune cells to eliminate viral infected cells might hyper-activate other immune mediators such as macrophage, neutrophils, monocytes, dendritic cells among others to release excessive cytokines to compensate for the low functional lymphocytes that might ultimately represent cytokine storm [45].

Infiltration of tumor infiltrating lymphocytes (TILs) are observed in different types of cancer including OC and has been significantly correlated with patient outcome (reviewed by [139]). The anti-tumor response or the tumor promoting response of these infiltrating immune cells is critically determined by the tumor microenvironment that constitute cytokines, chemokines, antigens and costimulatory molecules [49]. These immune cells can recognize specific antigens present on the surface of tumor cell and induce anti-tumor response [192]. However, cancer cells are also capable of creating an immunosuppressive microenvironment *via* the release of inhibitory cytokines, expression of inhibitory molecules and infiltration of immunosuppressive cells such as M2 macrophage and regulatory T cells (T Regs) [194] to down regulate anti-tumor activity of the lymphocytes. Characterization of lymphocytes in tumor and ascites from advanced stage OC showed higher proportions of CD4+ and CD8+ T cells expressing exhaustive markers such as PD1 and TIM-3 [151]. Additionally, the cancer patients undergoing treatment are immunocompromised and show lymphopenia. In the absence of the CD4+ and CD8+ T effector lymphocytes, which play a key role in eliciting antiviral responses [126] and dendritic cells, which play a major role in controlling antiviral interferon responses [137], vaccine may be functionally incompetent for a cancer patient [15]. Previous reports show that ovarian cancer patients undergoing chemotherapy cannot generate antibody response to inactivated influenza vaccines [28]. However, the data for efficacy and outcome of SARS-CoV-2 vaccines in ovarian cancer patients is unavailable. OC which is associated with several comorbidities such as metabolic diabetes mellitus and obesity have altered cytokine profile in patients resulting in a pro-inflammatory and pro-tumorigenic microenvironment [33]. This can further complicate the immune system response to the vaccines with potential adverse immunogenic as well as cytokine storm reactions with side effects and may pose a major challenge to any vaccination strategy. Thus, further studies to explore potential side effects of SARS-CoV-2 vaccines in ovarian cancer patients as well as to improve our understanding of molecular relationships between cancer and SARS-CoV-2 are necessary.

## Conclusion

In this review, we have attempted to delineate and summarize the impact of comorbidities, gene defects, and inflammatory milieu associated with OC and SARS CoV-2 infection. While there is merit for using agonists and antagonists of specific pathways as anti-cancer agents, their use in the clinical therapeutics requires caution. The network of inflammation-related genes modulated in SARS-CoV-2 infection and the underlying comorbid conditions may promote alterations in signaling pathways that could consequently lead to severe inflammation-induced cancer pathogenesis and/or impart undesirable outcomes in OC patients. Defining the immunological landscape of tumors upon SARS CoV-2 infection may facilitate implementation of effective anti-cancer therapy and disease management.

## Data Availability

Not applicable

## Abbreviations

OC: Ovarian cancer
EOC: Epithelial ovarian cancer
GLOBOCAN: Global cancer statistics
COVID-19: Coronavirus disease
SARS-CoV-2: Severe acute respiratory syndrome coronavirus 2
SARS-CoV: Severe acute respiratory syndrome coronavirus
MERS-CoV: Middle-East respiratory syndrome coronavirus
WHO: World Health Organization
S-protein: Spike glycoprotein
RAAS: Renin-angiotensin-aldosterone system
FSH: Follicle stimulating hormone
LH: Luteinizing hormone
GnRH: Gonadotropin releasing hormone
OCPs: Oral contraceptive pills
HRT: Hormone replacement therapy
TLR: Toll like receptors
HNP1: Human neutrophil peptide
IL: Interleukins
CCL2: CC-chemokine ligand 2
M-CSF: Macrophage colony-stimulating factor
TNF-α: Tumor necrosis factor α
TRAIL: Tumor necrosis factor (TNF)-related apoptosis inducing ligand
MIF: Macrophage migration inhibitory factor
LIF: Leukemia inhibitory factor
TNFR1: Tumor necrosis factor receptor 1
NF-κB: Nuclear factor kappa B
HIF1α: Hypoxia-inducible factor 1-alpha
STAT: Signal transducer and activator of transcription
CRP: C-reactive protein
ACE2: Angiotensin-converting enzyme 2
RIG-I/MDA-5-MAVS: Retinoic acid-inducible gene I /melanoma differentiation-associated gene 5- mitochondrial antiviral signalling protein axis
IRF: Interferon regulator factor
IFN: Interferon
ARDS: Acute respiratory distress syndrome
ALD: Acute lung Damage
TGF-β1: Transforming growth factor-beta 1
MCP: Monocyte chemoattractant protein
MAPK: Mitogen-activated protein kinase
ERK: Extracellular signal-regulated kinase
BK: Bradykinin
BKB1R: Bradykinin B1 receptor
BKB2R: Bradykinin B2 receptor
DABK: (des-Arg9)-BK
G-CSF: Granulocyte-colony stimulating factor
IP10: Interferon gamma-induced protein 10
SCF: Stem cell factor
HGF: Hepatocyte growth factor
bFGF: basic fibroblast growth factor
GRO-α: Growth-regulated oncogene alpha
MIG: Monokine induced by interferon (IFN)-γ
CTACK: Cutaneous T cell-attracting chemokine
SDF-1α: stromal cell-derived factor 1
MIP: Macrophage inflammatory protein
VEGF: Vascular endothelial growth factor
SCGF: Stem cell growth factor
TMPRSS: Transmembrane serine protease
PRL: Prolactin
RLRs: RIG1 like receptors
cGAS-STING: cyclic GMP-AMP synthase-stimulator of interferon genes
TBK1: TANK binding kinase 1
IKKε: Inhibitor of nuclear factor kappa-B kinase subunit epsilon
cGAMP: cyclic GMP-AMP
NSP: Non-structural protein
ACE1/Ang-II/AT1: Angiotensin-converting enzyme-1/angiotensin II/ angiotensin II type 1 receptor
ACE2/Ang1-7/ MAS: Angiotensin-converting enzyme-2/ Angiotensin (1-7)/ MAS1 proto-oncogene
NEP: Neutral endopeptidase
PEP: Prolyl endopeptidase
sIL-6R: Soluble interleukin 6 receptor
gp130: glycoprotein
Th: T helper
MPV: Mean platelet volume
C5b: Complement component 5b
MLB: Mannose-binding lectin
hCG: human chorionic gonadotrophin
SCD1: stearoyl-CoA desaturase-1
GTEx: Genotype-Tissue Expression
N: Nucleocapsid
VE-cadherin: Vascular endothelial-specific cadherin
IAV: Influenza A virus
PRLR: Prolactin receptor
PAMPs: Pathogen-associated molecular patterns
PlGF: Placental growth factor
PDGF-BB: Platelet-derived growth factor-BB
RANTES: Regulated on activation, normal T cell expressed and secreted
DAMPs: Damage associated molecular patterns
MHC I: Major histocompatibility class I
HPV: Human papillomavirus
VLP: Virus-like particles
PD-1: Programmed cell death protein 1
Tim-3: T-cell immunoglobulin mucin-3
TILs: Tumor infiltrating lymphocytes
T Regs: Regulatory T cells
